# Fostering school reintegration after psychiatric inpatient treatment: description and study protocol of an evaluation study about a rehabilitation program for children and adolescents with chronic school refusal (SchuTIng-stAR)

**DOI:** 10.3389/frcha.2025.1629877

**Published:** 2025-09-11

**Authors:** U. Neumann, V. Just, L. Henke, M. Knollmann, S. Zellmer, M. Andzinski, S. Schmidtendorf, M. Noack, M. Föcker, J. Seitz, M. Holtmann

**Affiliations:** ^1^ LVR-University Hospital Essen, Clinic for Child and Adolescent Psychiatry, Psychosomatics, and Psychotherapy, Essen, Germany; ^2^ The German Pension Insurance (DRV, Deutsche Rentenversicherung Westfalen), Münster, Germany; ^3^ LWL-University Hospital Hamm for Child and Adolescent Psychiatry, Ruhr-University Bochum, Hamm, Germany

**Keywords:** school refusal, emotionally based school absenteeism, rehabilitation, school reintegration, mental health, school attendance, study protocol, children and adolescents

## Abstract

**Background:**

School refusal among children and adolescents with mental health issues carries long-term risks for their educational trajectories, future employment, mental health, and social participation. Despite the availability of multiple treatment approaches, a significant number of adolescents continue to experience difficulties with school attendance following inpatient therapy or partial hospitalization. To enhance reintegration into school, a rehabilitation project called “educational participation and integration for children and adolescents with mental illness through a seamless stepwise rehabilitation program” (“SchuTIng-stAR”) was developed specifically for children and adolescents with severe and persistent school refusal associated with psychiatric disorders who are at risk of continued school attendance problems after psychiatric inpatient treatment or partial hospitalization.

**Methods/study design:**

After describing the therapeutic rationale, the development, and the content of the program, the study protocol for its evaluation using both quantitative and qualitative methods is presented. The primary objectives of the evaluation are firstly to assess the effects of the treatment on psychological symptoms and school attendance, and secondly to identify factors that influence the participation and engagement of patients, parents, and other stakeholders involved (teachers, youth welfare services). The operationalization of outcomes, measurement methods and hypotheses regarding effectiveness are described. Measurements will be taken at three points in time: at the beginning of the rehabilitation intervention (T1), at the end of treatment (T2) for the main outcome and after a six-month follow-up period (T3) for follow-up assessment. Therefore, it is a one-group pretest–posttest design with follow-up period. Additionally, it is explained how interviews with families will be analyzed using qualitative content analysis.

**Discussion:**

The formative and summative evaluation of innovative treatment programs for children and adolescents, including the perspectives of relevant stakeholders, is essential to ensure their sustainability and their integration into already existing services provided by health and social care systems. As chronic school avoidance is a multifactorial and complex condition and its course is often characterized by relapses, it is important to develop sustainable treatment approaches and to closely examine treatment commitment using qualitative methods. The discussion focuses on the extent to which the rehabilitation intervention and the study produce the expected results, and what factors might contribute to divergent outcomes.

## Introduction

### Background

Attending school ensures that children and adolescents not only gain cultural skills such as literacy and numeracy and acquire general knowledge but also develop social skills and key qualifications for their future professional and private lives. This is closely associated to the opportunity to lead a self-determined and independent life and to participate equally and actively in social life, what has been examined in detail for years in the OECD Education report “Education at a glance”^1^. The aim of compulsory schooling, which exists in Germany and several other countries, is to ensure basic education for all children, regardless of the social class, cultural background and financial status of their parents, and thus to enable employment and independence from social security systems.

**Table 1:** Treatment elements.

Treatment module	Professionals	Components of intervention
Pharmacological and psycho-therapeutic treatment	Psychiatrists, Psychotherapists	Medical examination, monitoring or initiation of pharmacological treatment, assessement of symptoms and school avoidance (functional analysis), psychoeducational sessions, goal setting, teaching skills for problemsolving in school situations, peer contact and family situations, preparations for the first day in school, support in maintaining motivation for change, special therapeutic interventions according to the psychiatric symptoms (CBT and/ or 3rd-Wave interventions, including Multi-family-treatment) ⇒ in individual and group therapy setting ⇒ with children/adolescents and/or parents/ families
Nursing and educational treatment	Nursing staff, educational staff	Support in everyday life, training of everyday skills like preparation for school (training for using public transport, for social skills and planning skills), support in establishing leisure activities, counselling for parents and families how to manage challenging situations, accompaniment to school
Social service	Social worker	Organization of participation conferences, establishing networks of support with other care systems for the families, home visits, connecting parents and school, advice for teachers about the psychiatric symptoms, accompaniment to school
Other therapies	Occupational therapistst, psychomotor therapists	Training of school and work-related skills, training of social skills, improvement of self-efficacy, concentration and planning skills
Participation conferences	Social worker, therapists, Pension insurance advisor, nursing and educational staff, teacher	Assessment and goalsetting, planning for the stepwise treatment, estabilshing of an interdisciplinary support system between (Rehabilitation project, school, therapists, familiy, youth welfare system), planing and monitoring the success, assessment for further need for support in family members
School	Teacher of clinic school	Training of school skills, preparation for first days at school, exchainging informations with the school

Knollmann et al. (1) demonstrate that beside the persisting problem of missing robust data about school absenteeism, we can expect that in Germany about 5%–10% of children and adolescents show problematic school absenteeism despite compulsory education. While absenteeism is minimal in primary school, it increases over the adolescent years (2–4). Beside other risk factors, school absenteeism is one significant factor of school dropout (5). Klemm (4) reports that 5.7–6.9% of German adolescents leave school without lower secondary school certificate, which seems to consist over several years. Rumberger (6) describes as a consequence of absenteeism, that these children and adolescents are at greater risk of being unemployed or employed in lower paid jobs (p. 151). So, it is more likely for them to face financial insecurity or poverty, become dependent on social security systems, to suffer from mental and physical health problems (1, 7–10) or to engage in criminal behavior (2, 6).

Reasons for school absenteeism are manifold (11–13) and various attempts have been made to categorize its different forms. A fundamental differentiation exists between truancy and school avoidance or school refusal. Truancy is typically defined as a deliberate absence from school, whereas school avoidance or school refusal results from emotional problems like anxiety, depression or bullying (13, 14). However, the terms are less clearly defined in the literature than it might initially appear (15, 16) and there are various criteria for subdivisions, such as function for basic needs or interpersonal relationships, school law or type of behavior (17). Havik et al. (13) outlines the careful clarification of the terms used to reveal and challenge potential preconceptions, such as equating truancy with intentional misconduct vs. recognizing school refusal as response to emotional distress. They clarify differences between “School Refusal Behavior (SRB)” as an “overarching construct” for “child-motivated” absence from school, “Truancy (TR)” as “unauthorized” or “illegitimate absence”, “School withdrawal (SW)” caused by parents and “School Refusal (SR)” which is behavior to avoid “strong negative emotions while” or “prior to school” (p. 3–4). Tamlyn (15) suggests the term “Emotionally based school absenteeism (EBSA)” to describe patterns of School Non-Attendance where children or adolescents are unable to attend school due to emotional reasons, although it is not very distinctive in regard of concepts like authorized/unauthorized, internal/external problems or psychiatric diagnoses. But he emphasizes that this group of children and adolescents requires special attention because of a high risk of chronicity (3, 17).

School non-attendance is particularly high among youth facing mental health difficulties, and there is a large number of children and adolescents who are affected by psychological problems. Reiß (18) reports prevalence rates of mental health problems and specific anxious and depressive symptoms among children aged 7–17 years in Germany in the range from 22% to 18% between 2003 and 2017. During the COVID-19 pandemic, which took place mainly in 2020 and 2021, these rates increased to as much as 30%. Although the prevalence of mental health problems subsequently declined, it remained elevated at 23% in the second half of 2022. Witte (19) demonstrates that especially adolescent girls have experienced a marked increase in anxiety and depressive disorders in 2022 compared to prepandemic levels of 2019. Lester (20) mentions an impact of increasing mental health issues on school attendance problems in England, which is likely to take place in Germany too. Unfortunately, we have no recent data until now.

Previous research shows, that school absenteeism has complex interacting causes in family environment, personality, school and peer context (12, 21). Therefore, intervention programs targeting EBSA should take into account multiple life contexts and integrate different therapeutic approaches. Melvin and colleagues (22), in their “Kids- and Teens at School framework (KiTeS)”, demonstrate that, from a bio-ecological perspective, interventions should also target broader macro-level factors by engaging stakeholders from education, health, and youth services sectors.

In the case of EBSA it is important to focus on therapeutic concepts that address the existing psychological symptoms. Previous treatment approaches have shown that both, psychological symptoms and school absenteeism can be improved (23–26). However, the specificity of school-avoidant behavior has not always been targeted specifically and accompanied reintegration into school is rare. This could be the reason for relapses or progression of EBSA (7).

While there are a few programs for reintegration in school after psychiatric hospitalization or severe mental health issues in the USA and in Canada [cf. Tougas et al. (27)], they are rare in Germany. White et al. (28) describe that in the USA the number of young people with severe mental health problems and the necessity for inpatient treatment is increasing whereas the length of the treatment is decreasing. Because of remaining symptoms after the treatment and because of challenges that emerge from the step back to school itself (e.g., fear of stigmatization, dealing with missed learning), there is need for help for reintegration.

Although rehabilitation, which aimed at restoring everyday life skills and work ability is available directly after inpatient treatment for physical illness [*“Anschlussheilbehandlung”* or early medical rehabilitation (29)] for example after neurological, orthopedic or oncological hospital treatment, there is no comparable offer after hospital treatment for mental health problems, especially not for children and adolescents and especially not focused on reintegration in school after times with school absenteeism before hospital treatment.

In the recent years it became obvious that increasing mental health problems (30) have an impact on many people's ability to participate in work and social life (30–32). In Germany in 2016 a law called the “Bundesteilhabegesetz” (BTHG) (33) was passed and the Federal Ministry of Labour and Social Affairs initiated a funding program called “rehapro”, which aims at fostering model projects to introduce and research new interventions for prevention and early intervention in case of participation restrictions in work and social life due to medical handicaps. In accordance with the above-mentioned significant correlation between EBSA in youth and later employability, this program includes treatment which address health-issues in childhood and youth.

Therefore, the LWL-University Hospital Hamm, the German Pension Insurance and the LVR-University Hospital Essen formed a working group to develop a new rehabilitation project for children and adolescents: “SchuTIng-stAR”—an educational participation and integration program that helps children and adolescents with mental illnesses by focusing on seamless and stepwise rehabilitation. This project is for 12–18-year-old children and adolescents, who had received inpatient treatment or partial hospitalization for mental health problems. It provides a rehabilitative care immediately following hospitalization. The objective of this project is to facilitate the full reintegration of children into school and to maintain this integration over time.

**Figure 1:**
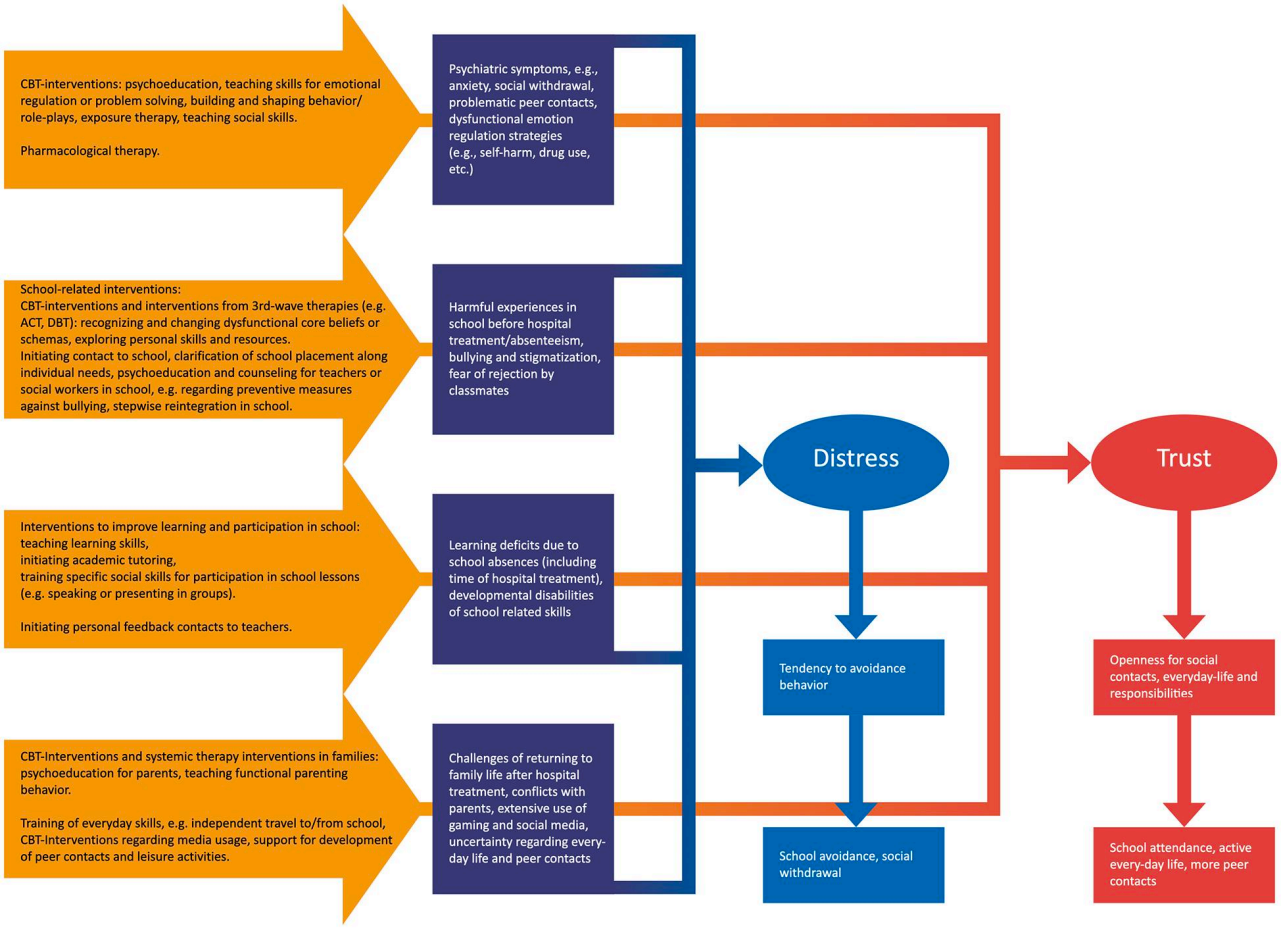
Theory model of the treatment. The blue boxes show the problems and following dysfunctional behaviour. The yellow arrows show the different interventions to address the problems and the red boxes the desired results.

Recognizing the multifactorial and multisystemic nature of EBSA, the rehabilitation project includes child, family, and school-focused interventions, as well as counseling for teachers and the youth service members involved. Based on an existing psychiatric treatment program for EBSA (25) a rehabilitation program was adapted and extended, which resulted in a rehabilitation project for sustaining school attendance and social participation of patients with chronic EBSA after inpatient treatment. Following a stepped-care-approach, it includes different intensity levels ranging from inpatient-, partial hospitalization-, and outpatient rehabilitation. Our focus is specifically on addressing withdrawal and avoidance behaviour resulting from psychological symptoms, previous stressful experiences, and the resulting dysfunctional perceptions or negative expectations regarding reintegration into school. By enhancing emotional regulation, social skills, academic learning, and everyday functioning, we aim to strengthen self-efficacy, in turn, foster greater openness and motivation to engage in social interaction, initiate activities, and develop confidence in managing everyday tasks such as attending school (see Figure 1).

**Figure 2:**
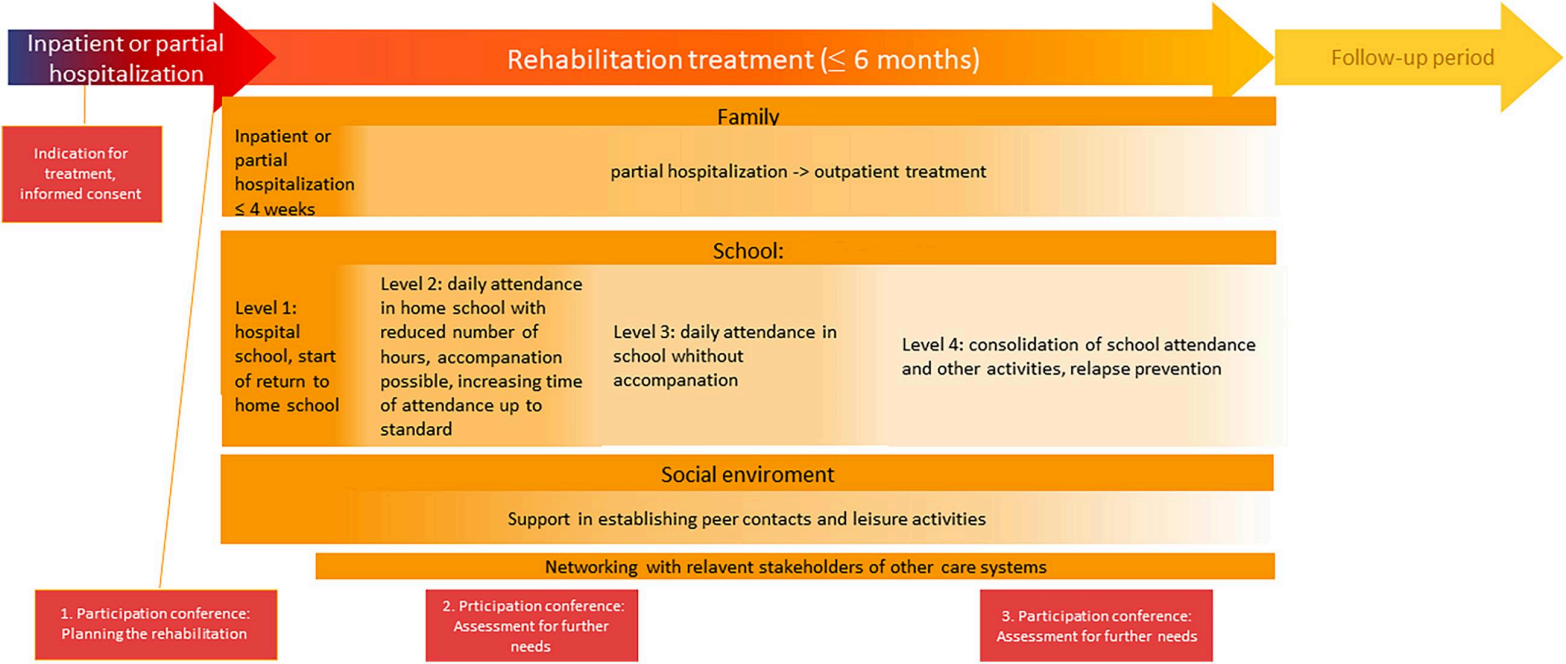
Treatment phases.

There are frequent problems with school attendance after the inpatient treatment, which families are not always aware of. Knollmann et al. (1) describe that up to one third of adolescents with mental health issues who searched for clinical treatment showed persisting problems with school attendance or vocational training after 1.5–3 years after the first consultation. Therefore, we have sought to direct the parents' and adolescents' attention to the importance of successful reintegration and a stable school attendance early in treatment. Thus, we implement a close exchange with the previous treatment team of inpatient or partial hospitalization and invite the families to an early information session. Once they have decided to participate in the rehabilitation project, a participation conference takes place. There, all the assistance systems—inpatient or day care treatment team, rehabilitation team, school, family, adolescents, possibly already working youth assistance representatives—will be involved in order to exchange information about the current symptoms and discuss the need for support. An individualized treatment plan for school reintegration will be developed, and further support can be assessed.

The treatment is composed of individual and group sessions, parents' talks and parents' groups, occupational therapy and psychomotor treatment, social service and everyday support from an educational-care team (see Table 1). In the first phase treatment is continued in the inpatient or day care setting, then the treatment shifts more and more into the family setting. Thus, a combination of treatment elements in the clinic and prospective support in the daily environment takes place. In this way both can be served: further continuation of treatment for the underlying mental health disorder and gradual transfer and reintegration into everyday life. This includes supporting young people in more active leisure activities but also enabling families to communicate better with each other and to cope with everyday family life. Support for parents in challenging situations is also possible.

In the post-care phase, there are more outreach services including telephone support for acute problems. Due to the focus on school reintegration, it is also possible to accompany the child to school by members of the rehabilitation team for some time, if necessary. Participation conferences take place three times to ensure the exchange of all actors involved and the individual adaptation of the treatment. If one compares this design of treatment with the framework for interventions addressing school reintegration from Tougas (34), it fits for almost all nine steps of the framework.

### Objective

The aim of this article is to present the study protocol for the evaluation of the described model project. On the one hand the evaluation study has a summative concern, by getting information whether the treatment can improve school attendance, reduce clinical symptoms and enhance participation of young people (main outcome) and whether these effects can be maintained in the long term (follow-up assessment).

The hypotheses are:

1. At the end of treatment psychiatric and school-related symptoms (e.g., anxiety, depression, problems with peers at school, problems with teachers) will improve, school attendance increases, and global functioning in daily live will improve, too.
2. The improvement could be maintained or even augmented in the following six months.

To this end, data on symptoms, school attendance and global functioning will be collected at three points in time: at the beginning of treatment, at the end of treatment and six months after the end of treatment. On the other hand, the formative evaluation aims to explore the acceptance of the treatment by the treated families, the treatment team and stakeholders. For this purpose, data will be collected from stakeholders and interviews will be conducted with children, adolescents, and their parents.

## Methods and analysis

There are three institutions involved: The LWL-University Hospital Hamm is responsible for the implementation of the rehabilitation, the German Pension Insurance (DRV-Westfalen), which is in charge of the project, ensures the structural integration into the guidelines of rehapro and rehabilitative care. They are also involved in the participation conferences mentioned above. The LVR-University Hospital Essen will conduct the evaluation study, which has been approved by the ethics committee of Ruhr-University-Bochum (to which the LWL-Clinic belongs) and the medical department of the University Duisburg-Essen. The study is registered in the German Register of Clinical Studies under the ID DRKS0032415.

### Study design

The presented study follows a mixed-methods approach: It is divided into a summative evaluation with quantitative data analysis and a formative evaluation with qualitative data analysis. The quantitative data analysis is based on a pre-post design with a six-month follow-up period. There is no control group; therefore, we have a prospective, non-randomized, non-blinded, single-arm pre-post design. Following Landes (35) and Curran (36) the study is a hybrid implementation-effectiveness study of type 1, because of both, interest and data collection in regard of effectiveness of the intervention and of information about factors that would facilitate participation for adolescents, parents and stakeholders.

Patients will be recruited from inpatient and partial hospitalization units of the LWL-University Hospital Hamm. If the clinical treatment team identifies any prognostic evidence of problems with school reintegration after hospital treatment—such as persistent preadmission absenteeism or a tendency to anxious-avoidant behavior, which can't be addressed enough by hospital treatment—they may register the patient for the rehabilitation project during ongoing clinical treatment. There are standardized criteria (see below) which has to be met.

The children or adolescents and their parents will have a thorough consultation with the responsible therapists of the rehabilitation team to receive detailed information about the rehabilitation project and the evaluation study.

There has to be a written consent from both, children and parents, to participate in the treatment and the study. The families get the information that they may be admitted to the rehabilitation project regardless of participation in the evaluation study.

Provided the adolescents and their parents give their consent, the teachers and stakeholders of other care systems involved will be informed about the study and asked for written consent in the course of treatment, as they are not always known at the beginning.

Prior to the main study, we will conduct a pilot phase with 15% of the calculated number of participants (=25 participants) to assess the feasibility and to adapt the treatment program to clinical conditions.

### Inclusion criteria

Inclusion to the study is possible when the following criteria are met:

1. The children and adolescents to be included in the project have to be 12–18 years old and receiving treatment due to a mental health problem according to ICD-10 in an inpatient or partial hospitalization unit of the LWL-University Hospital Hamm.
2. There has to be a period of at least 10% of time with school-absent behavior in the tree month before hospital treatment. This is calculated as the percentage of hours missed in relation to the number of lessons scheduled. Clinical therapists are instructed to ask this information by admission of every patient. Even if there has been no school attendance for weeks or months, patients could be included.
3. The need for rehabilitation and the ability to benefit from rehabilitation has to be assessed by clinical therapists or doctors. No ICD-10 diagnoses are excluded *a priori*, but there has to be a positive prognosis regarding the success of the treatment. Therefore, criteria like medical need, physical and psychological ability to participate, basic consent about the treatment goal (reintegration into school), need for support in daily life or other limitations to participate in society are checked. This means vice versa, that a lack of basic group skills (e.g., communication skills or acceptance of group rules), acute suicidal tendencies or aggressiveness toward other patients or caregivers are exclusion criteria.

If all inclusion criteria are met, the information will be passed to the rehabilitation team, in order to contact the families for further information approximately halfway through the ongoing treatment time. The inclusion will take place after the informed consent and the treatment contract have been signed. The beginning of the rehabilitation project coincides with the ending of hospital treatment. The patients remain in the same treatment unit of the LWL-University Hospital Hamm, but the rehabilitation team, which comes to the inpatient or partial hospitalization unit especially for the included patient, will replace the treatment team.

### Sample size and randomization

Our aim is to recruit a total of *N* = 162 children or adolescents for the rehabilitation project and the study. Based on an estimate of the number of adolescent patients which showed problems with school attendance and problems with reintegration into school after psychiatric hospital treatment in the LWL-University Hospital in Hamm in the year prior to the study, we arrived at a treatment figure of 162 for the duration of 24 month of the study. To every adolescent included in the study is assigned a code consisting of a sequential number. For the interviews, a random selection of thirty cases was made previously on the basis of the codes from 1 to 162. The number of 30 cases was chosen to ensure a representative range of experiences and for possible mixed-methods analysis. At the beginning, all families will be informed about the data collection.

### Exclusion criteria

Although the inclusion criteria are met, families may be excluded if their home or the school is too far from the clinic (more than 30 Kilometers). In this case the accompany to school or other outreach treatment elements are not possible. If acute crises or physical illness occur requiring inpatient treatment (e.g., suicidal crises), the rehabilitation may be interrupted for up to two weeks and then continued seamlessly. If inpatient treatment is required for a longer duration (more than 14 days), the rehabilitation project will be terminated.

Exclusion from the rehabilitation can also take place if the inclusion criteria are no longer met, e.g., if motivation for active participation in therapies can no longer be maintained. These cases will be included in the evaluation as long as the agreement is obtained.

### SchuTIng-stAR interventions

The rehabilitation project is parted into three phases with four treatment levels due to school attendance can be distinguished (see Figure 2).

First, there is a preparatory phase, which begins during the ongoing hospital treatment. It includes a consultation for information about the treatment, clarification of motivation, assessment for the including criteria and specification of treatment goals. As soon as the informed consent is signed, the first participation conference will take place where the multi-professional hospital treatment team, a representative of the DRV, the family, the adolescent and the multi-professional rehabilitation treatment team meet to specify treatment goals, special needs and treatment steps. On the same day as the end of the hospital treatment, the patient is admitted to the rehabilitation project.

At this point, the second phase of treatment starts. It continues in the previous setting (inpatient or partial hospitalization unit) and lasts a maximum of four weeks. Regarding school reintegration, the adolescents are at level one, where they either continue to attend the hospital school or can start with the return to home school. Besides establishing a therapeutic relationship with the adolescents, the focus is on psychoeducational elements and the concretization of treatment goals. Interventions based on CBT, including systemic therapy and of 3rd-wave therapy interventions, are used. It is offered in individual and group settings and targets still-existing mental symptoms—as well as building self-efficacy and resilience.

There is a home visit with the nursing staff and at least one therapeutic consultation with the parents as well as occupational therapy and psychomotor treatment. This is followed by the preparation for the return to the home school and the transfer to the home environment. After at least four weeks, the child is dismissed, transferred home, and the third phase of treatment starts, which includes level two to four of school reintegration.

In level two of school reintegration, the adolescents attend home school with a reduced number of hours. They can still be accompanied to school if necessary. The weekly therapeutic sessions in individual and group settings continue.

As soon as daily school attendance is possible without accompaniment, level three of school reintegration starts, which means that school attendance is gradually increased up to standard level and any remaining special arrangements at school are dismantled.

Level four focuses on strengthening the achievements in all areas (school, family, leisure activities) and intensive relapse prevention.

There will be two more participation conferences during the rehabilitation to assess further need for support. At this point, the completely family system is looked at to identify any need for support for other family members, which may affect the child's or adolescent's school attendance in rehabilitation.

### Data collection and survey instruments

The first data collection (T1) takes place at the beginning of rehabilitation. The Strength and Difficulties Questionnaire [SDQ^2^, (37, 38)] is used for the global assessment of mental symptoms first in the full-version and then in the follow-up version at the end of the rehabilitation (T2) and six months later (T3). This questionnaire is a very often used, short, easy and economical survey to assess emotional and behavioral problems. Although there are some subscales in the German version, which shows limited reliability, the total difficulties score demonstrates satisfactory psychometric properties, as well it shows overall good criterion and construct validity (39).

In addition, the Inventory of School Refusal [ISV (40)] is used at all three times (T1-T3), which consists of several scales measuring psychological symptoms and their impact on school attendance. A child version and a parent's version are available. We chose this instrument to get more information about school related symptoms. The evaluation study of Knollmann (40) showed good internal consistency and hints for discriminating validity to distinguish between symptoms.

For the individual attitudes and expectations towards further school attendance, the adolescents fill out the Self-Efficacy Questionnaire for School Situations (SEQ-SS^3^) (41) at all three time points (T1–T3). This questionnaire measures students' beliefs about their skills to manage different school situations. The reliability demonstrates good internal consistency (Cronbach's Alpha up to 0.83) and stable test-retest values (41). The surveys can be completed in the inpatient unit or at home, using a digital version implemented with the “Unipark”-tool.

To assess the level of functioning in daily life and activities, the Health of the Nations Outcome Scale for Children and Adolescents [HoNOS-CA (42)] is used, which can be completed by therapists, nursing staff or social workers after training. It's a short and easy-to-use instrument to assess overall psychosocial functioning from a professional perspective. Although there is heterogeneity in reliability and validity (43, 44) we decided to use it in our study to assess changes in everyday functioning. At T1 and T2, this is done face-to face, at T3 it is done after a home visit or a telephone call.

To assess school attendance, information is collected from the students themselves, their parents and their teachers. We ask for the number of scheduled teaching hours (*y*) and the number of absent hours (*x*) to calculate the percentage of absenteeism (*p* = *x*/y*100). At T1, we ask about the rate of attendance in the last three months before admission to the clinic, at T2 we ask about the rate in the last four weeks before the end of rehabilitation and at T3 we ask about the rate in the last four weeks before the measurement. In addition, we ask at T2 which proximal school level is being targeted (promotion to the next grade in the following school year or graduation) and at T3, whether it has been reached.

Further data, such as age, gender, type of school, duration of treatment, setting, and ICD-10 diagnosis are collected by interviewing the previous treatment team. Therefore, they receive the case-report form with several instructions for data collecting.

The questionnaires for the formal evaluation were designed based on key questions according to the object of research, which is to obtain information about the acceptance and active participation in the rehabilitation project. They consist of 20–27 items that can be answered on a 5-point Likert-scale. They address the organization of the project (information transfer, transparency, cooperation of the treatment team and equipment of rooms etc.), the evaluation of several elements (therapy sessions, care and education, involvement of parents/ teachers/ youth welfare services, implementation of the stepped-care approach, participation conferences) and the changes experienced in daily activities, school attendance, family life, leisure activities and peer contacts. In addition, participants are asked if they would recommend the rehabilitation project to others.

Based on the same key questions, an interview guide was developed for parents and children. The interviews are semi-structured, which means that questions can be explained or developed in more detail. To ensure the quality of the interviews, the interviewers will be trained through role-plays. The interviews will be conducted at the clinic where the rehabilitation takes place and will be done by members of the research team. The audios will be digitally recorded and then transcribed according to a fixed transcription guideline.

## Data analysis

### Quantitative data analysis

We will do statistical analysis, for example variance analysis with repeated measurement and chi-square tests to evaluate following hypotheses:

a) Main outcome: In regard of the development of school attendance and school related symptoms during treatment: The overall symptoms (SDQ) and the school related symptoms (SEQ-SS, ISV) will improve significantly from the beginning of the rehabilitation project to the end of the treatment (SDQ, ISV: T1 > T2; SEQ-SS: T1 < T2). At least 75% of the participants show a regular school attendance, defined as <10% of absenteeism in school weeks during the period T2—four weeks. The global functioning in daily life improves significantly from beginning to end of treatment (HoNOS-CA: T1 > T2).
b) Follow-up assessment: In regard of the development of school attendance and school-related symptoms after the treatment: The overall symptoms and the school-related symptoms will stabilize or improve in the 6 months following the end of treatment (SDQ, ISV: T2 ≥T3, SEQ-SS: T2 ≤ T3). At least 75% of the participants achieve the proximal school goal and at least 75% of the participants show a regular school attendance, defined as <10% absenteeism during the period T3—four weeks. The global functioning in daily life is maintained or improved in the six-month following the end of treatment (HoNOS-CA: T2 ≥ T3).

Data analyses will be performed with SPSS software.

### Qualitative data analyses

For the formative evaluation, we will need self-designed surveys, which ask for the formative evaluation. They will be evaluated using summative scores and cluster analyses.

The interviews are based on a semi structured guide: there are predefined questions that can be explored in more depth interactively during the interview. The questions relate to the decision to participate in the program, which treatment components were offered and participated in, whether these were helpful, and how the cooperation within the treatment team or between stakeholders was perceived. In addition, participants are asked whether their goals were achieved and what exactly helped them to achieve these goals. Finally, participants are asked to summarize their satisfaction with the treatment. The interviews are conducted by trained researchers and then transcribed verbatim.

The analysis of the interviews will use focused analysis of qualitative interviews (45). In this technique, relevant categories are identified through intensive reading of transcripts interviews, summarizing passages and abstracting, then building codes that are fine-tuned by applying them to transcripts of further interviews. The generated codes then are used to find hypotheses about several formative evaluation questions, e.g., do the parents and adolescents think that the rehabilitation project is helpful, which aspects are important for their opinion, which aspects are important for acceptance of the treatment, which aspects are likely to cause a premature end? Then the interrelationship between the results of the quantitative and the qualitative analyses will be discussed.

## Discussion

Because of the continuing high number of children and young people with mental illnesses (18, 19) and the associated fractures in their educational biographies with long-term effects (1, 4), there is an urgent need to improve existing treatments and develop new approaches (1, 12). This is the concern of the described rehabilitation project. The individual and societal costs of low educational attainment, chronic absenteeism and avoidance are long-lasting and high (6, 10). In Germany, the social security system with health, long-term care, unemployment and pension insurance funds the treatment of these restrictions. However, this implies that these systems should work as efficiently as possible. Therefore, a scientific evaluation of all community-financed interventions is of high interest. This study aims to contribute to this.

Previous research on existing treatment for children and adolescents with EBSA shows that there are many helpful approaches especially for the treatment of mental illness (23–28). However, focusing on sustainable school reintegration and promoting participation in all areas of daily life is still little widespread (46). If the scientific evaluation shows that SchuTIng-stAR is an appropriate intervention, which is acceptable to families and has an impact on school attendance and mental health, this may help to ensure that the offer is continued and possibly extended to other clinics.

However, there are limitations to the systematically derivation of causal effects due to the research design. First, there is no control group. This was decided in order to ensure that as many children and adolescents as possible could participate in the rehabilitation project.

Second, the intervention is a completely new form of treatment. We hope to minimize the need for adapting the treatment during the study period by piloting first. But there still might emerge some aspects which leads to adoption or sharpening of interventions during the trial period, what will be documented thoroughly. This also limits the value of the data to be extracted.

Nevertheless, it seems reasonable and worthwhile to evaluate this intervention in this explorative way in order to gain insight into outcome trends relevant factors. The mixed-methods design with interview data and qualitative analysis methods also addresses the need to gain insights in a treatment setting that is as natural as possible. In this way, we can learn about not only the reduction of school absenteeism, but also about what makes it easier for children, adolescents and families to participate in such an intervention, and what leads to treatment refusal, dropout or lack of success.

We are confident that the study, despite its methodological limitations, can make a valuable contribution to understanding the difficulties that mentally ill children and adolescents have in attending school. So wecan provide insights into the needs of support in the systems involved (family, school, youth welfare) to cooperate with each other in the best interests of the young people and to help to design effective treatment approaches.

## Footnotes

^1^ https://www.oecd.org/en/publications/serials/education-at-a-glance_g1gha6b0.html

^2^ https://www.sdqinfo.org/a0.html

^3^German version kindly provided from PD Dr. V. Reissner, Düsseldorf
